# Aortic Valvuloplasty for Incomplete Quadricuspid Aortic Valve Using Tricuspidization by Folding the Accessory Cusp

**DOI:** 10.1016/j.atssr.2025.08.018

**Published:** 2025-09-12

**Authors:** Atsuyuki Mitsuishi, Yujiro Miura, Kazuya Miyagawa, Takashi Kunihara

**Affiliations:** 1 Division of Cardiovascular Surgery, Department of Surgery, Kochi Medical School Hospital, Okohcho, Nankoku-shi, Kochi Prefecture, Japan; 2 Department of Cardiology and Geriatrics, Kochi Medical School Hospital, Okohcho, Nankoku-shi, Kochi Prefecture, Japan; 3 Department of Cardiac Surgery, The Jikei University School of Medicine, Nishishinbashi, Minato-ku, Tokyo, Japan

## Abstract

We describe a young patient with incomplete quadricuspid aortic valve, poor medication adherence, and multiple dental caries. Aortic valve repair was selected over replacement and successfully performed by tricuspidization, folding the accessory cusp toward the commissure between the right and noncoronary cusps while preserving the raphe. The postoperative course was uneventful, and mild aortic insufficiency persisted without progression at 2 years.

Quadricuspid aortic valve (QAV) is a rare congenital anomaly, and incomplete forms are even more uncommon. There is currently no consensus on the optimal surgical approach for incomplete QAV. This report describes a case of incomplete QAV with severe aortic regurgitation, successfully managed with a tailored tricuspidization technique.

We report the case of a 49-year-old man with hypertension, dyslipidemia, and chronic renal failure who presented to another hospital with exertional dyspnea (New York Heart Association class III) and was diagnosed with severe aortic insufficiency (AI) 10 months before surgery. Preoperative transesophageal echocardiography showed left ventricular diastolic/systolic diameter (LVDD/LVSD) of 63/41 mm, left ventricular ejection fraction (LVEF) of 64%, and no asynergy. The aortic valve appeared tricuspid with severe AI due to marked right coronary cusp (RCC) prolapse.

He had multiple dental caries and a cementum osteogenic fibroma in the right mandible. After oral surgery, he was referred to our department. Because of his age, poor adherence, and high risk for infectious endocarditis, repair was favored. Median sternotomy and cardiopulmonary bypass were performed through ascending aorta and right atrium cannulation. Antegrade cardioplegia was given every 30 minutes.

Intraoperative findings revealed an incomplete QAV (Figures 1a-1c), with an accessory cusp (AC) between the RCC and noncoronary cusp (NCC), contradicting the preoperative diagnosis. Regurgitation was due to RCC and AC redundancy, causing prolapse and bending without fenestration. The annulus measured 28 mm. Geometric/effective heights were as follows: left coronary cusp, 18/10 mm; RCC, 18/5 mm; NCC, 19/8 mm. Preoperative imaging showed the sinotubular junction measured 35 × 32 mm and the mid-ascending aorta measured 37 × 33 mm.

**Figure 1:**
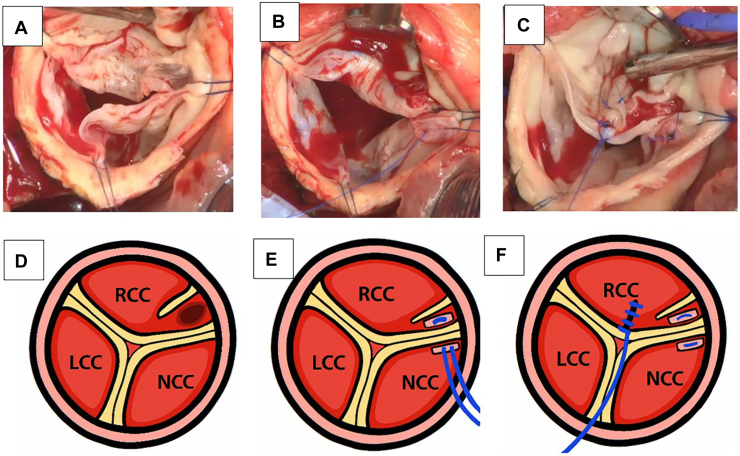
Intraoperative findings and schematic of valve repair. (a) Incomplete quadricuspid aortic valve with an accessory cusp and pouchlike structure. (b) Folding repair by suturing the pouchlike structure to the right coronary cusp (RCC)–noncoronary cusp (NCC) commissure using pericardial pledgets. (c) Central plication of the RCC free margin. (d-f) Corresponding schematics showing (d) pre-repair anatomy, (e) folding with buttress sutures, and (f) central plication. (LCC, left coronary cusp.)

Correction was achieved by folding redundant tissue with mattress sutures, preserving the AC raphe (Figures 1b, 1e), reducing the RCC free margin. External suture annuloplasty with CV-0 (GORE-TEX; Gore Medical) reduced annular diameter to 20 mm. Central plication of the RCC was added (Figures 1c, 1f).

The ascending aorta was replaced above the sinutubular junction with a 22-mm Dacron graft (J Graft; Japan Lifeline). Aortoscopy showed slight NCC shortening, requiring central plication. Final inspection confirmed good cusp geometry (Figure 2).

**Figure 2:**
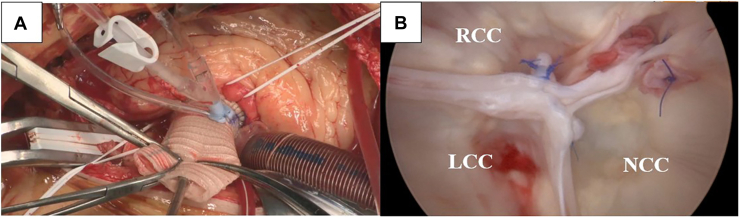
Intraoperative view and aortoscopic assessment after valve repair. (a) Intraoperative view showing completion of aortic valve repair and ascending aortic replacement with a prosthetic graft. (b) Postrepair aortoscopic view demonstrating a competent tricuspidized aortic valve. (LCC, left coronary cusp; NCC, noncoronary cusp; RCC, right coronary cusp.)

Intraoperative transesophageal echocardiography showed trivial AI and mean pressure gradient of 3 mm Hg. Cardiac arrest and cardiopulmonary bypass times were 117 and 140 minutes. On postoperative day 7, transthoracic echocardiography showed LVDD/LVSD of 51/43 mm, LVEF of 38%, and mild AI (Figure 3), with pressure gradient of 4 mm Hg. He was discharged on day 25. At 1 year, transthoracic echocardiography showed LVDD/LVSD of 47/29 mm, LVEF of 69%, and mild AI. At 2 years, LVDD/LVSD was 47/28 mm and LVEF was 72%, with persistent mild AI.

**Figure 3:**
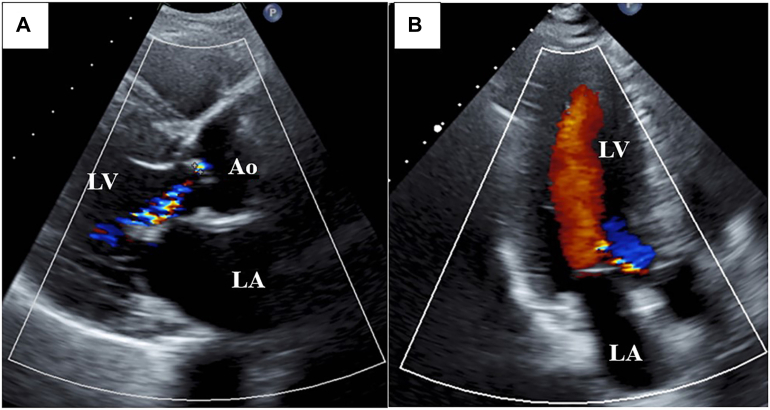
Postoperative transesophageal echocardiography. (a) Long-axis view showing mild residual aortic insufficiency on color Doppler imaging. (b) Continuous-wave Doppler across the aortic valve demonstrating a mean pressure gradient of 8 mm Hg. (Ao, aorta; LA, left atrium; LV, left ventricle.)

## Comment

The prevalence of QAV is <0.5%.^1^ The progression of cusp fibrosis, leading to failure of cusp coaptation, is proposed as the main underlying mechanism of AI.^1^ Given the patient’s age, poor medical adherence, and elevated risk for infectious endocarditis, valve repair was chosen over replacement. It also reduces prosthetic-related complications and improves long-term outcomes in infectious endocarditis.^2^ In general, bicuspidization is considered a stable option in cases with 4 equal-sized cusps or with 2 large and 2 small cusps, where commissural geometry allows symmetric 180-degree reconstruction. In contrast, QAVs with 3 normal cusps and 1 small AC, such as in our case, are more commonly managed with tricuspidization.^3^ However, our case did not fit neatly into any existing classifications, as the quadricuspid valve was incomplete, with one hypoplastic accessory cusp, a morphology that is not represented in the Hurwitz and Roberts classification.^4^ Borjian and Hali^5^ reported that QAV may result from incomplete partitioning of a cusp during embryogenesis or fusion with a raphe between 2 primary cusps (AC and NCC). In this case, the primary issue was prolapse of the RCC and AC. Tricuspidization was first attempted by folding the raphe into the RCC-NCC commissure, which compressed the AC but distorted the RCC. We then folded the AC while preserving the raphe to the RCC-NCC commissure using mattress sutures with pericardial pledgets. This reduced the RCC free margin and corrected both RCC and AC prolapse. Correcting the height discrepancy with sutures and adjusting the free margin by central plication appears to be a practical approach. Although cases with calcification or sclerosis of the AC are generally excluded, we believe that if the AC exhibits adequate pliability and continuity of the free margin, tricuspidization incorporating the AC is feasible using the same surgical principles, regardless of its location or size.

Ascending aortic replacement was chosen because an anastomosis above the sinutubular junction allows reliable reduction to 22 mm. This ensures uniform cusp reduction, and double suture annuloplasty is a dependable technique,^6^ although not covered by insurance in Japan. Using a prosthetic graft as a ring substitute and proceeding with replacement was deemed appropriate. The physiologic annular diameter is 21.5 ± 2.0 mm in adult men with a body surface area of 1.8 m^2^.^7^ In this case, with a body surface area of 1.79 m^2^, either 20 mm or 22 mm was suitable. A 20-mm Hegar dilator was used, and a 22-mm graft was selected, corresponding to a 1-size increase when considering the annulus to sinutubular junction ratio of 1:1.1 to 1.2.^8^ No postoperative aortic stenosis was observed.

In summary, we encountered a rare case of incomplete QAV characterized by an AC with a pouchlike structure and associated prolapse of the RCC. Because of poor medical compliance and oral hygiene, valve repair was chosen. Tricuspidization, achieved by folding the AC toward the RCC-NCC commissure while preserving the raphe, successfully corrected AI. The patient remains well at 2 years, although longer follow-up is needed to assess durability.

## Declaration of Generative AI and AI-Assisted Technologies In The Writing Process

During the preparation of this work, the authors used ChatGPT, an AI language tool developed by OpenAI, to improve language and readability. The final version was reviewed and approved by the authors.

## References

[1] Tsang M.Y., Abudiab M.M., Ammash N.M. (2016). Quadricuspid aortic valve: characteristics, associated structural cardiovascular abnormalities, and clinical outcomes. Circulation.

[2] Mayer K., Aicher D., Feldner S., Kunihara T., Schäfers H.J. (2012). Repair versus replacement of the aortic valve in active infective endocarditis. Eur J Cardiothorac Surg.

[3] Schmidt K.I., Jeserich M., Aicher D., Schäfers H.J. (2008). Tricuspidization of the quadricuspid aortic valve. Ann Thorac Surg.

[4] Hurwitz L.E., Roberts W.C. (1973). Quadricuspid semilunar valve. Am J Cardiol.

[5] Borjian S., Hali R. (2022). A quadricuspid aortic valve with incomplete partitioning can mimic a tricuspid aortic valve. J Cardiovasc Echogr.

[6] Youssefi P., Brega C., Shraer N., Zacek P., Debauchez M., Lansac E. (2019). Isolated bicuspid aortic valve repair with double annuloplasty: how i teach it. Ann Thorac Surg.

[7] Capps S.B., Elkins R.C., Fronk D.M. (2000). Body surface area as a predictor of aortic and pulmonary valve diameter. J Thorac Cardiovasc Surg.

[8] De Kerchove L., Momeni M., Aphram G. (2018). Free margin length and coaptation surface area in normal tricuspid aortic valve: an anatomical study. Eur J Cardiothorac Surg.

